# Adrenergic signaling and oxidative stress: a role for sirtuins?

**DOI:** 10.3389/fphys.2013.00324

**Published:** 2013-11-08

**Authors:** Graziamaria Corbi, Valeria Conti, Giusy Russomanno, Giancarlo Longobardi, Giuseppe Furgi, Amelia Filippelli, Nicola Ferrara

**Affiliations:** ^1^Department of Medicine and Health Sciences, University of MoliseCampobasso, Italy; ^2^Department of Medicine and Surgery, University of SalernoSalerno, Italy; ^3^Doctoral School of Translational and Clinical Medicine, University of SalernoSalerno, Italy; ^4^Fondazione S. Maugeri, Istituto di TeleseTelese Terme, Italy; ^5^Department of Medical Translational Sciences, Federico II University of NaplesNaples, Italy

**Keywords:** oxidative stress, sirtuins, GRK2, β-adrenergic system, exercise training, heart failure, reactive oxygen species

## Abstract

The adrenergic system plays a central role in stress signaling and stress is often associated with increased production of ROS. However, ROS overproduction generates oxidative stress, that occurs in response to several stressors. β-adrenergic signaling is markedly attenuated in conditions such as heart failure, with downregulation and desensitization of the receptors and their uncoupling from adenylyl cyclase. Transgenic activation of β2-adrenoceptor leads to elevation of NADPH oxidase activity, with greater ROS production and p38MAPK phosphorylation. Inhibition of NADPH oxidase or ROS significantly reduced the p38MAPK signaling cascade. Chronic β2-adrenoceptor activation is associated with greater cardiac dilatation and dysfunction, augmented pro-inflammatory and profibrotic signaling, while antioxidant treatment protected hearts against these abnormalities, indicating ROS production to be central to the detrimental signaling of β2-adrenoceptors. It has been demonstrated that sirtuins are involved in modulating the cellular stress response directly by deacetylation of some factors. Sirt1 increases cellular stress resistance, by an increased insulin sensitivity, a decreased circulating free fatty acids and insulin-like growth factor (IGF-1), an increased activity of AMPK, increased activity of PGC-1a, and increased mitochondrial number. Sirt1 acts by involving signaling molecules such P-I-3-kinase-Akt, MAPK and p38-MAPK-β. βAR stimulation antagonizes the protective effect of the AKT pathway through inhibiting induction of Hif-1α and Sirt1 genes, key elements in cell survival. More studies are needed to better clarify the involvement of sirtuins in the β-adrenergic response and, overall, to better define the mechanisms by which tools such as exercise training are able to counteract the oxidative stress, by both activation of sirtuins and inhibition of GRK2 in many cardiovascular conditions and can be used to prevent or treat diseases such as heart failure.

## Introduction

The sympathetic adrenergic system plays a central role in stress signaling and stress is often associated with increased production of reactive oxygen species (ROS).

ROS production is the result of several mechanisms, including generation during oxidative phosphorylation in the mitochondria as a product of normal cellular aerobic metabolism (Davies, 1995; Ide et al., 1999). Thus, the major process from which the body derives sufficient energy can also result in the production of ROS (Ide et al., 1999). The balance between the production of ROS and the activation of the antioxidant defense system is crucial for the human physiology and the control of cellular homeostasis. ROS play an important role in signaling processes, but their overproduction generates oxidative stress. In fact, ROS can regulate cellular functions, e.g., during immune and inflammatory processes (Remacle et al., 1995), in turn their overproduction causes damage to cellular constituents, including DNA, proteins, and lipids, especially when occurs with insufficient antioxidant enzyme activity (Varma, 1991).

In several cellular signaling pathways (Nishida et al., 2000), ROS act as second messengers downstream of specific ligands, including Transforming Growth Factor-β1 (TGF-β1), Platelet-Derived Growth Factor (PDGF), Fibroblast Growth Factor-2 (FGF-2), endothelin (Thannickal and Fanburg, 2000; Sawyer et al., 2002; Griendling and FitzGerald, 2003; Machida et al., 2003) and they are also involved in modulating the activity of specific transcription factors, such as Nuclear Factor-kB (NF-kB) and Activator Protein–1 (AP-1) (Hsu et al., 2000; Hirotani et al., 2002; Turpaev, 2002; Wu et al., 2002; Sabri et al., 2003; Rengo et al., 2013a).

Elevated ROS have also been implicated in the development and sustainment of several chronic degenerative diseases (i.e., cancer, diabetes, neurodegenerative and cardiovascular conditions) and in the mechanism of senescence and aging (Knight, 2000; Dröge, 2002; Westerheide et al., 2009; Marciano et al., 2012; Paolillo et al., 2013; Rengo et al., 2013b), and it has been suggested that they also contribute to adverse myocardial remodeling and the progression to heart failure (Sawyer et al., 2002; Seddon et al., 2007). However, relatively little is known about the type of ROS involved (e.g., superoxide, hydrogen peroxide, peroxynitrite), their specific role in mediating myocyte hypertrophy, apoptosis, fibrosis that participate in myocardial remodeling (Sawyer et al., 2002; Mann and Bristow, 2005; Seddon et al., 2007), and in relationship to the overall progression to myocardial failure.

The sympathetic adrenergic system plays a central role in ability to rapidly respond to various types of threats. One important target of adrenergic stimulation is the heart, where activation of β-adrenergic receptors causes increases in heart rate (chronotropy), relaxation speed (lusitropy) and contractility (inotropy) (Andersson et al., 2011).

Increased adrenergic drive is a major factor influencing the development of pathological cardiac hypertrophy, a stage which precedes overt heart failure. Whereas it is well known that heart failure, a highly prevalent syndrome, is characterized by both increased ROS production and β-adrenergic hyperactivity, still few evidence are available on the relationship between β-adrenergic system and oxidative stress.

Recently it has been discovered that a family of enzymes consists of NAD^+^-dependent histone/protein deacetylases, called Sirtuins, represents pivotal regulator of redox cellular status.

In mammalian cells SIRT1 appears to control the cellular response to stress by regulating the family of Forkhead transcriptional factors (FOXOs) (Brunet et al., 2004) and directly deacetylating the Heat Shock Factor (HSF1) and thus regulating Heat Shock Proteins (HSPs) expression (Westerheide et al., 2009; Corbi et al., 2012a).

This review is aimed to focus on the relationship between adrenergic system activity and oxidative stress, with a light on the possible implications of sirtuins in the regulation of this mechanism.

## Oxidative stress in the cardiovascular system

Several *in vitro* and *in vivo* studies have demonstrated ROS activation in the cardiovascular system in response to various stressors and in the failing heart (Ide et al., 1999; Cesselli et al., 2001; Wallace, 2001; Sawyer et al., 2002; Sabri et al., 2003; Scortegagna et al., 2003; Suematsu et al., 2003), and animal studies have also suggested that antioxidants and ROS defense pathways can ameliorate ROS-mediated cardiac abnormalities (Chen et al., 1996; Yen et al., 1996; Ho et al., 1998; Conrad et al., 2004; Giordano, 2005).

The ROS oxide (O2-), nitric oxide (NO), hydroxyl (OH-), and peroxynitrite (ONOO-) are molecules characterized by the presence of unpaired electrons that are highly reactive with cysteine residues in the catalytic center of cellular enzymes, thus making them excellent signal transducers (Finkel, 1999).

ROS have been linked to key pathologic processes such as cardiac hypertrophy (Nakamura et al., 1998) cardiomyocyte apoptosis (von Harsdorf et al., 1999), ischemia-reperfusion (Zweier et al., 1989) and heart failure itself (Ide et al., 1999). But also oxidant overproduction occurs in response to several stressors, including chemicals, drugs, pollutants, high-caloric diets, and exercise (Kohen and Nyska, 2002). Physical exercise can increase oxidative stress, eventually causing a perturbation of homeostasis that is dependent on training specificity (Conti et al., 2012a) and workload (Conti et al., 2013), but in turn it is also able to counterbalance the deleterious effects of ROS by activation of several antioxidant systems, such as Super Oxide Dismutases (SODs), HSPs and catalase (Corbi et al., 2012a,b). The mechanisms by which ROS mediate these different biologic responses are not fully understood, but in many cases involve activation of specific redox-sensitive signaling molecules. Three important candidates for downstream effectors are p38 Mitogen-Activated Protein Kinase (p38MAPK) and c-Jun Kinase (JNK), members of the stress-activated kinase family, and the cell survival kinase Akt (Griendling et al., 2000).

Angiotensin II, Tumor Necrosis Factor alpha (TNF-α) and norepinephrine are neurohormones implicated in the development of cardiac hypertrophy and progression to end-stage human heart failure (Packer, 1998). There is currently evidence that at least some hypertrophic effects induced by these agents are mediated through ROS.

*In vivo*, under physiologic conditions, O2- is predominantly inactivated by SODs, which are present in high concentrations in mitochondria (MnSOD), cytosol (Cu/Zn SOD), or plasma membrane/extracellular spaces, and consequently the formation of ONOO- is minimal.

During physiological and pathological conditions, including aging, SODs convert O2- to hydrogen peroxide (H_2_O_2_), which has a longer half-life, can diffuse longer distances than O2-, and is able to influence signaling events at more distant sites. In fact H_2_O_2_ can regulate the activity of several enzymes essential for Ca^2+^ release, growth, or apoptosis (phospholipases A2, C, and D, Src kinase, p38MAPK, JNK and Akt/PKB) (Griendling et al., 2000).

It has been demonstrated that the expression and activity of the SOD system is modified in aging, with reduced cell ability to counteract the oxidant molecules, and consequent weak resistance to ROS accumulation (Rinaldi et al., 2006). Obviously, cytotypes with limited replication ability, such as brain and heart, are particularly vulnerable to this phenomenon, suggesting that it could explain, at least in part, high prevalence of cardiovascular and neurological disorders in elderly people (Navarro-Arévalo et al., 1999). In fact, it is widely known that oxidative stress and reduced antioxidant defense have negative effects on cardiac structure and function (Singal et al., 1988) and they are also involved in lipid membrane oxidation and other heart age-related conditions (Corbi et al., 2012b).

HSPs are another system of cellular defense against oxidative stress. These “stress-induced proteins” are ubiquitous and highly conserved chaperones, important in the folding of new synthesized or damaged proteins. Moreover, HSPs mediate mitochondrial protection against oxidative stress and some of those, such as HSP70, have been associated with myocardial protection. Martin et al. (Martin et al., 1997) showed an increased survival in HSP70-transfected cardiomyocytes and consequent increased expression of the HSP70 enzyme against ischemic cardiac damage.

The metabolism of H_2_O_2_ is tightly regulated by the cellular glutathione peroxidases, which scavenge H_2_O_2_ (glutathione-dependent) or catalase (glutathione-independent) (Sorescu and Griendling, 2002). By converting H_2_O_2_ into water, catalase constitutes a primary antioxidant defense system and could protect cells from ROS and its deleterious consequences on diseases.

Recently it has been demonstrated that catalase protected cardiac mitochondrial aconitase enzyme from oxidative damage (Schriner et al., 2005) and overexpression of catalase targeted to mitochondria protects mice from cardiac aging, providing direct evidence for the role of mitochondrial ROS in the aging of this vital organ (Dai et al., 2009).

In fact, accumulation of oxidative damage has also been considered responsible of many different aspects of the aged heart. It has been found that cardiac fibrosis and size of myocytes increase with aging, while the number of myocytes decreases and ventricular hypertrophy is almost a constant finding in the aging rat heart (Anversa et al., 1986; Klima et al., 1990; Besse et al., 1994a). Hearts of old rats are characterized by reduced antioxidant defenses, such as SODs and Hsp 70 (Rinaldi et al., 2006).

Moreover, the oxidative stress with abnormalities in mitochondrial function, calcium (Ca^2+^) handling, electrolytes alterations, hormones, and cardioprotective signaling have all been proposed as potentially implicated in the aging process (Besse et al., 1994b). In particular, regarding the effects of electrolytes changes implicated in the regulation of myocardial function, it has been demonstrated that magnesium (Mg^2+^) interferes on failed cardiac contractility (Corbi et al., 2008) by modifying sarcoplasmic reticular Ca^2+^ transport systems with a calcium antagonism mechanism based on competition between Mg^2+^ and Ca^2+^ for the same binding sites on key myocardial contractile proteins, such as troponin C, myosin, and actin (Koss and Grubbs, 1994) that could explain the opposite effects of Mg^2+^ and Ca^2+^ on myocardial contractility (Kawano, 1998). Ca^2+^ overload can be induced by direct effect of ROS on Ca^2+^ handling proteins or indirectly, by inducing membrane lipid peroxidation (Valko et al., 2007).

## Sirtuins and oxidative stress in the cardiovascular system

Another important mechanism involved in cellular redox regulation is represented by family of sirtuins, a cluster of seven homologous proteins regulating cellular biology and metabolism through deacetylation of histones and other cellular factors such as NFkB, HSF1, p53, FOXOs, and Peroxisome Proliferator-Activated Receptor Gamma Coactivator (PGC-1). By promoting deacetylation, sirtuins can either promote or inhibit the activity of several protein targets (Finkel et al., 2009; Haigis and Sinclair, 2010; Guarente, 2011).

SIRT1 and SIRT6 can deacetylate specific lysines on histone tails to promote transcriptional silencing. SIRT1 also deacetylates many non-histone proteins such as p53, FOXOs, Nuclear Receptor Corepressor (SMRT/NCOR), and PGC-1alpha (Finkel et al., 2009; Haigis and Sinclair, 2010; Guarente, 2011). SIRT3 targets mitochondrial enzymes involved in metabolism, ROS detoxification, and mitochondrial function including Long Chain Acyl coe-enzyme A Dehydrogenase (LCAD), Isocitrate Dehydrogenase 2 (IDH2), SOD2, and cyclophilin D (Hafner et al., 2010; Zhong and Mostoslavsky, 2011). Other enzymatic reactions catalyzed by selected sirtuins are the transfer of an ADP-ribosyl group from NAD^+^ to an acceptor protein (SIRT1, SIRT4, and SIRT6) (Finkel et al., 2009; Haigis and Sinclair, 2010; Guarente, 2011), or the demalonylation and desuccinylation of modified proteins (SIRT5) (Du et al., 2011). However, the biological relevance of these reactions is only beginning to be unveiled (Oellerich and Potente, 2012).

In particular, SIRT1, the human homologous of the family, is involved in many functions of human physiology, including DNA repair, cell cycle regulation, apoptosis, gene expression, and aging (Grubisha et al., 2005). By FOXO3 acetylation and/or phosphorylation oxidative stress induces arrangement of SIRT1-FOXO3a, complex indispensable for cell cycle arrest and induction of DNA repair (Brunet et al., 2004). In turn, SIRT1 can modulate the cellular stress response directly deacetylating some proteins and regulating their expression (Porcu and Chiarugi, 2005). In fact, SIRT1 modulates the threshold of cell death in the setting of exogenous stress, including oxidative damage, interacting with p53, inhibits Bax-induced apoptosis by deacetylation of Ku70, and regulation of other targets linked to cell death (Cohen et al., 2004) and cellular antioxidant activity (such as Mn-SOD and catalase) (Corbi et al., 2012b).

Moreover, SIRT1 protects against endothelial dysfunction by preventing stress-induced premature senescence, thereby modulating the progression of cardiovascular diseases (Ota et al., 2007; Li et al., 2011; Nadtochiy et al., 2011; Stein and Matter, 2011), and it plays an essential role in mediating the survival of cardiac myocytes under stress *in vitro* (Alcendor et al., 2004; Pillai et al., 2005).

It has been observed that overexpression of Sirt1 reduces expression of the Angiotensin II Type 1 Receptor (AT1R) (Sunagawa, 2008) and this inhibition seems to prevent endothelial dysfunction of cerebral arterioles (Arrick et al., 2008; Miyazaki et al., 2008).

Although there are fewer studies of the other sirtuins, the importance of SIRT3 for cardiac function has been demonstrated by some authors. SIRT3 is expressed abundantly in the heart, and has been reported to play a protective role against hypertrophy, acting at different levels. SIRT3 overexpression blocks hypertrophy both *in vitro* and *in vivo*, whereas SIRT3^−/−^ mice exhibit enhanced susceptibility to hypertrophy (Sundaresan et al., 2009), likely indirectly protecting against cardiac hypertrophy by specifically control of ROS levels. Moreover, SIRT3 attenuates Hypoxia-Inducible Factor 1-alpha (HIF-1α) activity indirectly by controlling intracellular ROS (Finley et al., 2011), suggesting a central regulatory function of sirtuins in the cellular response to hypoxia (Oellerich and Potente, 2012).

More recently Cardus et al. demonstrated that the presence of SIRT6 in endothelial cells protects from telomere and genomic DNA damage, thus preventing a decrease in replicative capacity and the onset of premature senescence. These findings suggest that SIRT1 and SIRT6 collaborate at different levels to maintain endothelial homeostasis, with SIRT6 regulating chromatin functions and DNA repair, and SIRT1 intracellular signaling networks (Cardus et al., 2013).

Finally SIRT7 seems to be an essential regulator of tissue homeostasis in the heart through its interaction with p53. Sirt7-deficient primary cardiomyocytes show an approximately 200% increase in basal apoptosis, and a significantly reduced resistance to oxidative and genotoxic stress (Vakhrusheva et al., 2008; Corbi et al., 2013).

Because the sirtuins activity depends on NAD^+^ availability it has been suggested that their enzymatic activity is directly linked to the energy and cellular redox status via the NAD^+^/NADH ratio. Among the seven sirtuins, SIRT1 and SIRT3 are crucially involved in regulation of cardiomyocyte energy metabolism, production of ROS and signaling relevant to cell death/survival (Tanno et al., 2012) playing different roles in regulation of energy production and oxidative stress. Hearts consume large amounts of O_2_ and yield high levels of ROS in the mitochondria. In addition, various extracellular factors, such as angiotensin II and Tumor Necrosis Factor-alpha (TNF-alpha), induce ROS formation and promote cardiomyocyte death together with the mitochondrial ROS (Giordano, 2005).

It has been demonstrated that MnSOD is required for normal biological function of tissues. In fact, Li et al showed that Mn-SOD^−/−^ homozygous mutant mice die within the first 10 days of life with a dilated cardiomyopathy, accumulation of lipid in liver and skeletal muscle, and metabolic acidosis, and these findings were related to a severe reduction in succinate dehydrogenase (complex II) and aconitase (a TCA cycle enzyme) activities in the heart (Li et al., 1995).

Furthermore, Loch et al found that MnSOD^+/−^ mice displayed a decrease in fraction shortening and ejection fraction and an increase in left ventricular internal diameter in systole, and developed heart hypertrophy with accompanying fibrosis and necrosis, demonstrating that lifelong reduction of MnSOD activity has a negative effect on normal heart function (Loch et al., 2009).

Both SIRT1 and SIRT3 up-regulate Mn-SOD expression through different mechanisms, such as HIF-2a (Dioum et al., 2009) and/or FOXO4 (van der Horst et al., 2004) for SIRT1 and FOXO3a for SIRT3 (Sundaresan et al., 2009). Sundaresan et al. (2008) demonstrated that overexpression of both nuclear and mitochondrial SIRT3 protected cardiomyocytes from genotoxic stress and oxidant stress.

However, sirtuins adopt several other different tools to counterbalance the oxidative stress.

For instance, Alcendor et al. showed that overexpression of either Sirt1 or constitutively active FoxO1a in cultured cardiac myocytes stimulated expression of catalase, suggesting that FoxO1a plays an important role in mediating Sirt1-induced upregulation of catalase, which may in part mediate suppression of myocardial damage caused by oxidative stress (Alcendor et al., 2007).

SIRT3 also increases activity of other ROS-detoxifying enzymes indirectly. SIRT3 deacetylates and activates IDH2 and glutamate dehydrogenase in murine liver (Alcendor et al., 2007; Lombard et al., 2007), both of which produce NADPH in the mitochondria. NADPH in turn is required for glutathione reductase to convert oxidized glutathione to reduced glutathione, which is a crucial cofactor for mitochondrial glutathione peroxidase to scavenge ROS.

Shinmura et al. (2011) demonstrated that treatment of cardiomyocytes with resveratrol, an activator of SIRT1 and SIRT3, decreased ROS production and improved cell survival after hypoxia/reoxygenation without increasing the expression level of MnSOD protein.

Recently, mitochondrial ALdehyde DeHydrogenase 2 (ALDH2) has been identified as a novel target of SIRT3 (Schlicker et al., 2008; Lu et al., 2011). Excessive ROS in stressed hearts triggers lipid peroxidation and accumulation of reactive aldehydes, which in turn impairs mitochondrial function and induces cell damage. ALDH2 removes the aldehydes reducing the toxicity (Chen et al., 2010). Then, SIRT3-mediated ALDH2 activation could be another mechanism that mitigates cardiomyocyte damage induced by ROS, resulting in cardioprotection (Tanno et al., 2012).

## β adrenergic system and oxidative stress in cardiovascular system

It is well established that β-adrenoceptor (βAR) activation stimulates adenylyl cyclase activity through the participation of G proteins and promotes the formation of cAMP in the myocardium (Stiles et al., 1984; Bristow et al., 1990; Bohm, 1995; Chakraborti et al., 2000). The elevated level of cAMP increases the intracellular concentration of Ca^2+^ in cardiomyocytes on protein kinase A (PKA) mediated phosphorylation of different Ca^2+^-handling proteins in the membrane and produces the positive inotropic effect in the heart (Stiles et al., 1984; Bristow et al., 1990; Bohm, 1995; Chakraborti et al., 2000). This βAR-mediated signal transduction mechanism not only regulates the contractile activity of the healthy heart, but it is also considered to provide critical support for the maintenance of cardiac function during the development of heart failure (Bristow et al., 1990; Bohm, 1995; Post et al., 1999; Chakraborti et al., 2000; Sethi et al., 2007; Cannavo et al., 2013a).

In failing hearts, elevated sympathetic activity initially compensates for decreased cardiac contractility. βAR-mediated signaling is markedly attenuated in heart failure subjects, owing to the downregulation and desensitization of the receptors and their uncoupling from adenylyl cyclase (Rockman et al., 2002; Di Lisa et al., 2011; Rengo et al., 2012a; Femminella et al., 2013).

Many different mechanisms are implicated in the genesis of heart failure. Effects of high levels of insulin on the cardiovascular function are well studied. In a model of isolated rats papillary muscles, it was demonstrated that insulin-induced modulation of contractility is calcium independent and that insulin leads to a supersensitization on the β1-adrenoceptors (β1-AR) (Ferrara et al., 2005). At the same time, elevated plasma free fat acid levels have a stimulatory effect on sympathetic nervous system, as showed by decreased QTc interval after weight loss (Corbi et al., 2002; Bianco et al., 2013).

One of the pathophysiological mechanisms involved in the genesis of heart failure is represented by a persistent β1-AR stimulation, that evokes a multitude of cardiac toxic effects, including myocyte apoptosis and hypertrophy, as showed *in vivo* on rodent hearts and *in vitro* on cultured cardiomyocytes (Ferrara et al., 1997; Zheng et al., 2005; Cannavo et al., 2013b).

In particular it has been demonstrated a β1-AR downregulation and desensitization due apparently to overt and sustained stimulation, with largely unaltered β2-adrenoceptors (β2-AR) (Molenaar et al., 2007; Feldman et al., 2008; Rengo et al., 2012b), leading to an increased β2:β1 ratio.

β2-AR play an important role in the regulation of the angiogenic response in HF, as showed by the evidence that β2-AR overexpression was associated with a markedly increased capillary and arteriolar length density and enhanced *in vivo* myocardial blood flow and coronary reserve (Rengo et al., 2012b) and β-blockade promotes cardiac angiogenesis in heart failure via activation of VEGF signaling pathway (Rengo et al., 2013a).

G protein-coupled receptor kinases (GRKs) regulate numerous G Protein-Coupled Receptors (GPCR) by phosphorylating the intracellular domain of the active receptor, resulting in receptor desensitization and internalization. GRKs also regulate GPCR trafficking in a phosphorylation independent manner via direct protein-protein interactions (Evron et al., 2012).

GPCR are seven-transmembrane receptors that transmit a wide range of extracellular stimuli into cells, regulating the majority of biological processes. Upon agonist stimulation, GPCR activate G proteins, which exchange bound GDP for GTP, leading to the dissociation of the G protein into activated Gα and Gβγ subunits. This dissociation promotes downstream signaling through specific effector proteins and second messengers (Pierce et al., 2002; Takeda et al., 2002).

GRK2 is the most ubiquitous member of the GRK family. GRK2 rapidly phosphorylate GPCR upon agonist stimulation and facilitate the binding of arrestins to the phosphorylated receptors, leading to uncoupling of the receptor from the G protein (Pitcher et al., 1998a). This process, known as receptor desensitization, is the loss of receptor responsiveness upon prolonged stimulation. GPCR that are known substrates of GRK2 include the β2-AR, the chemokine receptors CCR2b and CCR5, the Platelet Activating Factor Receptor, and the neurokinin-1 receptor for substance P (Pitcher et al., 1998a; Lombardi et al., 2002).

GRK2 binds PhosphoInositide 3-Kinase (PI3K) and recruits it to the cell surface upon ligand stimulation of the βAR (Naga Prasad et al., 2001). This interaction has been shown to be important for βAR endocytosis, most likely via enhanced recruitment of AP2 to the receptor (Naga Prasad et al., 2002; Salazar et al., 2013).

Blocking the interaction of GRK2 with PI3K improves contractile function during heart failure by reversing βAR desensitization abnormalities and restoring βAR signaling (Perrino et al., 2005; Evron et al., 2012; Rengo et al., 2012c).

Interestingly, changes in GRK2 levels have been reported in a number of disease states. In human GRK2 levels are increased in myocardial tissue during heart failure (Ungerer et al., 1994; Rengo et al., 2013a,b,c), myocardial infarction (Santulli et al., 2011) and hypertension (Gros et al., 1997; Santulli et al., 2013).

Production of ROS has been detected in several cells stimulated with cytokines, peptide growth factors, and agonists of GPCRs (Thannickal and Fanburg, 2000). During inflammatory processes, lymphocytes are exposed to H_2_O_2_ and other ROS that are derived from activated macrophages and neutrophils as a first line of defense against invading pathogens. Further downstream, ROS regulate transcription factors, including NF-kB (Schreck et al., 1991).

**Figure 1:**
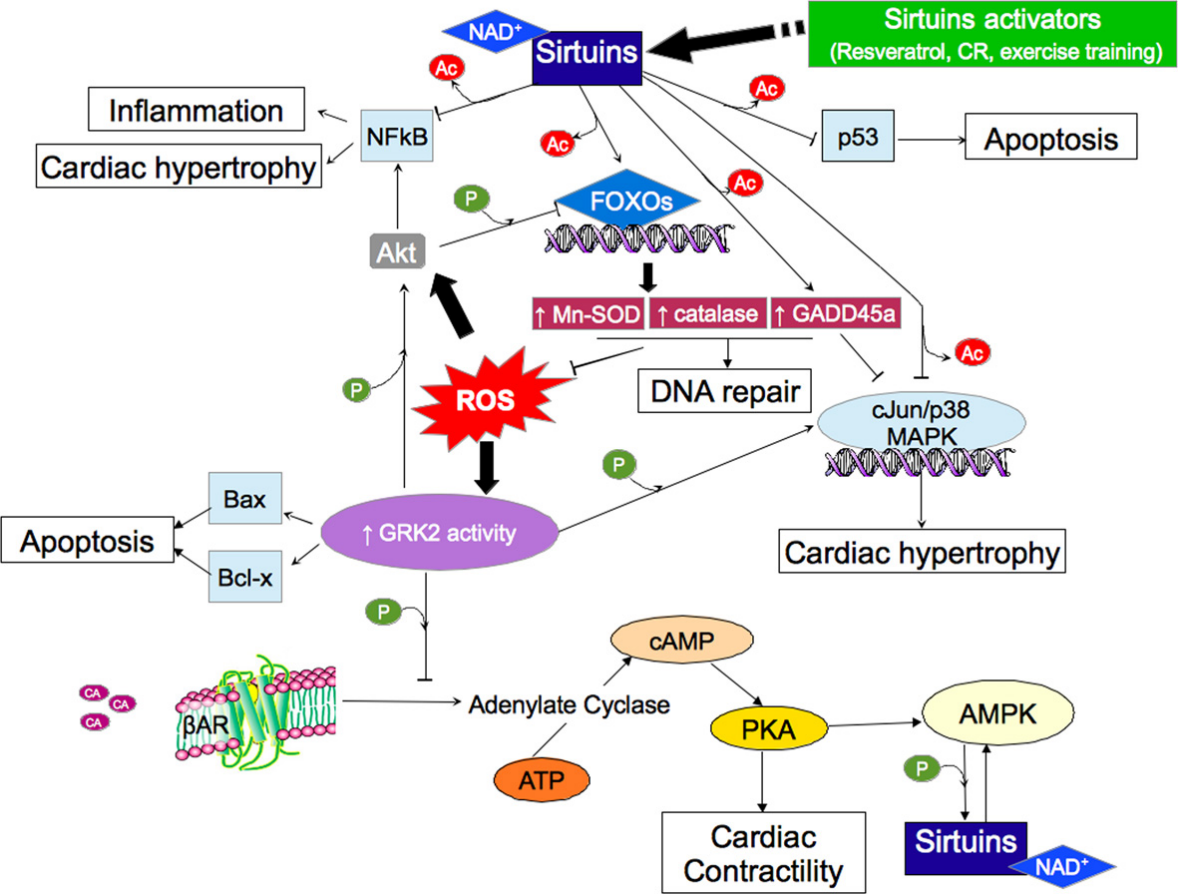
**Cellular response to oxidative stress mediating by β-adrenergic response and sirtuins involvement.** The ROS induce GRK2 hyperactivity that determines desensitization and internalization of β ARs with induction of cardiac hypertrophy (via AKT/NFkB pathway), apoptosis and senescence (by inhibition of FOXOs). Sirtuins (and their activators) are able to counteract these actions by direct effects on different molecules. ROS, reactive oxygen species; CR, caloric restriction; Ac, acetyl; P, phosphoryl. ↓ activation; ⊥ inhibition.

Some authors showed that exposure of lymphocytes to oxidative stress results in a decrease in cellular GRK2 protein levels. ROS produced by activated macrophages and neutrophils can alter the activity of lymphocytes. Exposure of lymphocytes to ROS results in increased intracellular calcium level, rapid tyrosine phosphorylation of a variety of proteins (Schieven et al., 1993), and activation of transcription factors such as NF-kB (Schreck et al., 1991; Lombardi et al., 2002) (Figure 1).

Oxidative stress activates several other kinase signaling pathways, such as Protein Kinase C (PKC), MAPK, and PI3K. Activated PKC can phosphorylate GRK2, with increased kinase activity (Chuang et al., 1995). Interestingly, inhibition of PKC does not affect basal GRK2 levels nor does it interfere with the H_2_O_2_-induced decrease in cellular GRK2. In addition, specific inhibitors of MAPK or PI3K do not have any effect on H_2_O_2_-induced decreases in GRK2 protein (Lombardi et al., 2002).

Previous reports showed that GRK2 is the predominant GPCR kinase involved in agonist-induced receptor sequestration of the β2-AR. Moreover, studies in transfected cell systems suggest that changes in the intracellular level of GRK2 alter the rate and extent of sequestration of the β2-AR (Ferguson et al., 1996; Penela et al., 1998; Lombardi et al., 2002).

A growing body of evidence has shown that GRK2 is capable of phosphorylating non-receptor substrates. GRK2 is a microtubule-associated kinase that directly phosphorylates tubulin following βAR stimulation (Pitcher et al., 1998b; Yoshida et al., 2003), suggesting a functional link between GRK2 and the cytoskeleton. Accordingly, GRK2 levels can affect agonist-induced βAR internalization in a mechanism involving microtubule stability (Vroon et al., 2007). GRK2-mediated phosphorylation of the membrane-cytoskeleton linkers, radixin (Kahsai et al., 2010) and ezrin (Cant and Pitcher, 2005), provides another indication for this functional link to the cytoskeleton.

Another important target of GRK2 kinase activity is the Insulin Receptor Substrate 1 (IRIS).

It has been reported that increased GRK2 levels mediate insulin resistance in myoblasts and adipocytes via a mechanism which involves sequestration of Gαq and IRIS (Usui et al., 2005; Garcia-Guerra et al., 2010). Interestingly, GRK2 directly phosphorylates IRIS in cardiomyocytes, a process that negatively affects cardiac glucose uptake and insulin sensitivity following ischemic injury and ultimately leads to the development of heart failure (Ciccarelli et al., 2011; Evron et al., 2012).

In fact, GRK2, also known as βAR kinase 1 (βARK1), provides a link between altered vascular/tissue physiology in insulin resistance and impaired IRIS signaling. GRK2 can interfere directly with Gαq/11-mediated signaling via its regulator of G protein signaling domain/GAP activity (Usui et al., 2004). Increased plasma concentration of the vasoconstrictive ET-1 polypeptide is associated with insulin resistance and/or hypertension (Kohno et al., 1990), which is, in turn, promoted by direct and indirect (sympathoadrenal and angiotensin II dependent) effects of compensatory hyperinsulinaemia to cause sodium retention (Yatabe et al., 2010). The correlation between excessive β-adrenergic activity and insulin resistance has long been noted (Deibert and DeFronzo, 1980). While tissue GRK2 levels have been correlated with plasma norepinephrine/epinephrine levels (Cho et al., 1999), GRK2 can be upregulated in cultured cells by chronic insulin (Garcia-Guerra et al., 2010), potentially as a result of PI3K-dependent stabilization of GRK2 (Salcedo et al., 2006). Thus, both local/circulating GPCR ligands associated with insulin resistance/hyperinsulinaemia, and insulin itself, contribute to the high GRK2 levels observed in insulin-resistant rodent/human tissues (Garcia-Guerra et al., 2010; Copps and White, 2012).

These findings demonstrate that lowering GRK2 in myocytes after ischemic injury will contribute to restore cardiac metabolism and prevent the development of subsequent heart failure (Evron et al., 2012).

Moreover, during heart failure GRK2 is up-regulated in the adrenal medulla, causing α2-adrenoceptor dysfunction and catecholamine hypersecretion. By decreasing GRK2 levels in the adrenal gland, β-blocker treatment appears to restore adrenal α2-AR density and signaling at the plasma membrane and catecholamine feedback inhibition, reducing sympathetic overdrive in chronic heart failure (Rengo et al., 2012c).

Thus, the favorable effects of GRK2 inhibition in cardiac disease can be ascribed not only to the direct improvement of adrenergic response but also to more complex interactions among different and specific systems involved in the pathophysiological response to myocardial injury (Rengo et al., 2012a).

Also it is well known that oxidative stress represents an underlying mechanism involved in insulin resistance development. The evidence that reactive nitrogen and oxygen species generation occurs when endothelial cells respond to high glucose (Garcia Soriano et al., 2001) suggests another link between oxidative stress and β-adrenergic activity in the involvement of many cardiovascular conditions.

ROS may change the functioning of GPCRs during disease processes via the calpain-dependent regulation of cellular GRK2 levels (Lombardi et al., 2002).

Moreover, *in vitro* studies have revealed several non-classical signaling molecules utilized by β2-AR, including β-arrestin 1 (Drake et al., 2008; Gong et al., 2008; Tilley et al., 2009), p38MAPK (Gong et al., 2008; McAlees and Sanders, 2009) and ROS (Yin et al., 2006; Gong et al., 2008). Transgenic activation of β 2-AR in cardiomyocytes leads to a sustained elevation of NADPH oxidase activity, which is accompanied by a greater ROS production as well as phosphorylation of p38MAPK. Inhibition of NADPH oxidase or ROS significantly reduced the p38MAPK signaling cascade. Chronic β2-AR activation *in vivo* is associated with greater extent of cardiac dilatation and dysfunction as well as augmented pro-inflammatory and profibrotic signaling, while antioxidant treatment protected hearts against these abnormalities, indicating ROS production to be central to the detrimental signaling of β2-AR. These findings highlight that the coupling of β2-AR with NADPH oxidase derived ROS/p38 MAPK is pivotal to the adverse signaling mechanism, and thus forms a potential therapeutic target (Xu et al., 2011).

More recently Chen et al. (2013) have been demonstrated that GRK2 localizes to heart mitochondria and it was an absolute requirement for prodeath signaling after oxidative and ischemic stress. Specifically, mitochondrial targeting of GRK2 in myocytes after ischemic injury promotes prodeath signaling because mitochondrial accumulation of GRK2 in myocytes increases after oxidative stress and it is dependent on ERK-mediated phosphorylation of GRK2, with subsequent movement to mitochondria dependent on binding of phosphorylated GRK2 to Hsp90. Then the authors suggested that blocking this mechanism led to cardioprotection.

## β-adrenergic system, oxidative stress and sirtuins

It has been demonstrated that sirtuins, NAD^+^/NADH deacetylases, are involved in modulating the cellular stress response directly by deacetylation of some factors that are also implicated in endothelial function control (Tang et al., 2012; Conti et al., 2013).

Sirt1 extends the lifespan of many organisms by increasing cellular stress resistance (Brunet et al., 2004; Alcendor et al., 2007), by an increase insulin sensitivity, a decrease circulating free fatty acids and insulin-like growth factor (IGF-1), an increased activity of the energy-sensing enzyme, AMP-activated Protein Kinase (AMPK), increased activity of Peroxisome proliferator activated receptor-gamma coactivator-alpha (PGC-1a), and increased mitochondrial number (Opie and Lecour, 2007). The requirement of NAD^+^ for Sirt1 activity implies that Sirt1 effectiveness depends on the cellular metabolic state (Conti et al., 2012a). Moreover, Sirt1 acts by involving signaling molecules such phosphatidyl-inositol-3-phosphate-kinase (PI3K)-Akt, MAPK (Bezstarosti et al., 2006) and p38-MAPK-β (Das et al., 2006) (Figure 1).

SIRT1 has been demonstrated to be localized predominantly in the nucleus or cytoplasm depending on the cell type. SIRT1 shuttles between the two cellular compartments in response to cellular stress in C2C12 cells and cardiomyocytes (Tanno et al., 2010), and during differentiation in neural precursor cells (Hisahara et al., 2008).

The nucleo-cytoplasmic shuttling is regulated by nuclear localization signals and nuclear export signals in the aminoacid sequences of SIRT1. PI3K/Akt- and JNK1- mediated phosphorylation of SIRT1 induces its nuclear translocation (Tanno et al., 2007; Nasrin et al., 2009). Nuclear localization of SIRT1 seems to be necessary for its protective function in cardiomyocytes (Tanno et al., 2007, 2010) whereas the biological significance of cytoplasmic SIRT1 remains to be determined. It has been demonstrated that resveratrol, a SIRT1 activator, improves insulin sensitivity in diet-induced obesity in mice (Baur et al., 2006; Lagouge et al., 2006). Sun et al. (2007) found that SIRT1 repressed protein phosphatase 1B (PTP1B) and thereby increased the level of insulin receptor phosphorylation, improving insulin sensitivity both in C2C12 myotubes and in high fat-fed mice.

Recently it has been demonstrated that βAR stimulation antagonizes the protective effect of the Akt pathway that is mediated by both insulin and hypoxia preconditioning, through inhibiting their induction of Hif-1α and Sirt1 gene, which are key elements in cell survival (Rane et al., 2010).

Akt overexpression in mice suppressed autophagy, which was associated with cardiac hypertrophy, interstitial fibrosis and contractile dysfunction (Hua et al., 2011). SIRT1 regulates autophagy by interacting with and deacetylating autophagy-related proteins Atg5, Atg7, and Atg8 (Lee et al., 2008). Recently, Hariharan et al. (2010) demonstrated that SIRT1 was required for starvation-induced autophagy in cardiomyocytes, in which SIRT1-mediated deacetylation of FOXO1 and subsequent activation of Rab7 plays a role.

Furthermore, FOXO1 was indispensable for maintenance of cardiac function after starvation, suggesting that autophagy induced by activation of the SIRT1-FOXO1 axis is an important adaptive mechanism in the failing heart (Tanno et al., 2012). Moreover, recently it has been demonstrated that reduced SERCA2a protein level, ventricular dysfunction, ventricular dilatation and mortality in a mouse model of type-1 diabetes were nearly normalized by treatment with resveratrol in a SIRT1-dependent manner (Sulaiman et al., 2010; Tanno et al., 2012).

The presence of high levels of norepinephrine has been considered as a pathological marker of heart failure (Tavares et al., 2008). Another demonstration of the relationship between adrenergic system and sirtuins is represented by the evidence that resveratrol prevents norepinephrine induced hypertrophy in adult rat cardiomyocytes, by activating NO-AMPK pathway (Thandapilly et al., 2011). Thandapilly et al. (2011) proposed that norepinephrine binds with the β-adrenergic receptor on the cardiac cell membrane, the sarcolemma, and activates phospholipase C resulting in the formation of 1,2-diacylglycerol (DAG) and inositol triphosphate (IP3). In turn, DAG stimulates cytosolic protein kinase activity resulting in increased protein synthesis leading to the development of cardiac hypertrophy (Eskildsen-Helmond et al., 1997).

In addition, resveratrol restored sirtuin activity, and thereby improve cardiac function in rats with diabetic cardiomyopathy (Sulaiman et al., 2010). Breen et al. (2008) studied the interaction between AMPK and sirtuin in resveratrol mediated signaling in skeletal muscle cells. In this study increased skeletal muscle glucose uptake was observed upon resveratrol treatment which was mediated by the sirtuin-AMPK dependent pathway (Breen et al., 2008). Moreover, it has been also demonstrated that resveratrol prevented cardiomyocyte hypertrophy by restoring the impaired AMPK activity in phenylephrine exposed cardiomyocytes as well as in SHR rats (Chan et al., 2008; Dolinsky et al., 2009) suggesting an important role for AMPK in mediating resveratrol effects.

Some authors (Dolinsky et al., 2009; Thandapilly et al., 2010) have recently reported that resveratrol prevented the development of pathological cardiac hypertrophy in genetically hypertensive rats without any effect on blood pressure, which is considered a pathological stimulus for the development of hypertrophy (Thandapilly et al., 2010, 2011).

The antioxidant activities of sirtuins are well known. SIRT3 blocks the cardiac hypertrophic response through activation of Foxo-dependent antioxidants, MnSOD and catalase, as well as suppressing ROS-mediated Ras activation and the downstream MAPK/ERK and PI3K/Akt signaling pathways (Sundaresan et al., 2009) (Figure 1). In particular, SIRT1 and SIRT3 appear to share similar ROS-accumulating end-point targets that cause cardiac hypertrophy. All of these findings support the hypothesis that use and development of sirtuin-specific activators and inhibitors may help further dissect the collaborative functions of SIRT1 and SIRT3 in the heart.

Less is known about the physiological role of SIRT7 in the heart. SIRT7 is a nuclear protein that associates with rDNA and interacts with RNA (Ford et al., 2006). It is not clear whether SIRT7 exhibits NAD^+^-dependent deacetylase activity, but reports suggest that it does respond to metabolic conditions by stimulating ribosomal biogenesis in dividing cells (Michishita et al., 2005) and it regulates heart cell death and damage by inhibiting p53, Ras, and Akt signaling pathways (Vakhrusheva et al., 2008). In fact, SIRT7-deficient mice develop heart hypertrophy and inflammatory cardiomyopathy, which is characterized by extensive fibrosis (Vakhrusheva et al., 2008). However, the molecular details explaining how SIRT7 targets these pathways remains unclear (Schug and Li, 2010).

Recently it has been proposed that β-adrenergic activation of the cAMP/PKA pathway rapidly increases SIRT1 activity in a NAD^+^ independent fashion. This mechanism enables SIRT1 to respond swiftly to the changing metabolic needs of the organism in settings of environmental stress. Cantó and Auwerx suggested that SIRT1 acts as a metabolic effector, synchronizing metabolic pathways with nutrient availability (Cantó and Auwerx, 2012). The molecular mechanism by which NAD^+^ regulates SIRT1 catalytic activity, however, is still not fully understood. In a low energy state, SIRT1 deacetylates and increases the activity of PGC-1a, leading to transcriptional upregulation of genes involved in lipid catabolism and mitochondrial biogenesis (Rodgers et al., 2005; Lagouge et al., 2006; Gerhart-Hines et al., 2007). Current understanding of the regulation of this process has emphasized a role for AMPK signaling in controlling the abundance of the SIRT1 substrate NAD^+^. The elevated AMP/ATP ratio during energy deficiency triggers phosphorylation of PGC-1a by AMPK, which primes PGC-1a for SIRT1-dependent deacetylation (and activation) (Cantó et al., 2009).

AMPK also increases the concentration of intracellular NAD^+^, further fueling SIRT1 deacetylase activity. However, both of these processes occur over the course of several hours, too long to permit rapid response to acute changes in energy stress.

SIRT1 activity has been reported previously to be regulated by post-translational modifications, such as phosphorylation by JNK. But the fact that the residues targeted by this pathway do not reside in the catalytic domain and are not conserved evolutionarily indicates this is an unlikely mechanism to regulate the well-conserved metabolic functions of SIRT1 (Cantó and Auwerx, 2012). Given the evidence supporting a function for SIRT1 in bioenergetics stress, Gerhart-Hines et al. (2011) hypothesized that stress-induced β-adrenergic signaling might regulate SIRT1 activity. Activation of the βAR increases intracellular cAMP concentration and activates PKA and its downstream effectors. The authors demonstrated that each component of the βAR-cAMP-PKA axis is essential to SIRT1 deacetylation of PGC-1a. In U2OS cells, treatment with β-adrenergic agonists (epinephrine and clenbuterol) or cAMP mimetics (forskolin and 8-BrcAMP) led to potent dose-dependent deacetylation of PGC-1a within 30 min. Forskolin-induced PGC-1a deacetylation was dependent on both PKA and SIRT1, but this effect was abolished by genetic deletion of SIRT1 in mouse embryonic fibroblasts. Furthermore, reduction of SIRT1 expression prevented forskolin-mediated upregulation of the PGC-1a target genes ERRa and PDK4 (Gerhart-Hines et al., 2011).

The rapidity with which SIRT1 transduced cAMP/PKA signals suggested that SIRT1 might be a direct target for PKA phosphorylation. Using mass spectrometry, the authors identified a residue on SIRT1 in the catalytic domain that was uniquely phosphorylated in response to forskolin treatment. They showed that Serine 434 (S434) phosphorylation was dependent on cAMP/PKA signaling and was rapidly reversed by removal of cAMP mimetic (forskolin). Furthermore, the authors showed that S434 phosphorylation was essential for the forskolin-induced increase in intrinsic SIRT1 enzymatic activity (Gerhart-Hines et al., 2011). Importantly, in all experiments, total NAD^+^ content was unchanged by cAMP/PKA signaling, indicating that cAMP-mediated deacetylation of PGC-1a was independent of NAD^+^ regulation of SIRT1. Gerhart-Hines et al. depicts SIRT1 as a dynamic orchestrator of both acute stress (βAR/cAMP signaling) and sustained energy crisis (AMPK-mediated changes in PGC-1a phosphorylation and NAD^+^ concentration) (Gerhart-Hines et al., 2007, 2011; Chao and Tontonoz, 2012).

## Conclusions

Oxidative stress represents the *primum movens* of several chronic degenerative diseases, especially of the cardiovascular system (Ferrara et al., 2006; Conti et al., 2012b). In the last decades several studies have demonstrated as the β-adrenergic system represents the target of the oxidative damage and, in turn, the responsible of oxidants production. The sirtuins, a new class of histone-deacetylases, seem to be the best defense of the cell to counterbalance the oxidative stress through the action on different pathways. Most part of the research on these molecules in the last years has been focused on the sirtuins activators, showing as the caloric restriction, the resveratrol and in particular the exercise training are able to mediate their beneficial effects by induction of sirtuins activity.

More recently, the use of a SIRT1 activator SRT2104 on cardiovascular function provided positive effects on lipid profiles, but were unable to demonstrate beneficial effects on vascular, endothelial, or platelet function compared with placebo (Venkatasubramanian et al., 2013).

Therefore, as suggested by Merksamer et al. (2013), for the future it will be important to develop experimental models in which the levels of oxidative stress and the activities of sirtuins can be precisely modulated to determine if sirtuins have a causative role in lifespan extension.

Moreover, as discussed above and showed in Figure 1, whereas the mechanisms involved in the cellular response to oxidative stress are represented by the same actors, very few studies have been performed to link the β-adrenergic system and sirtuins activity, and most of them are only focused on the metabolic pathway.

Therefore, more studies are needed to better clarify the involvement of sirtuins in the β-adrenergic response and, overall, to better define the mechanisms by which tools such as exercise training are able to counteract the oxidative stress, by both activation of sirtuins (Ferrara et al., 2008) and inhibition of GRK2 (Rengo et al., 2010) in many cardiovascular conditions.

The activation or overexpression of sirtuins leads to measurable increases in health and resistance to different stress, making them an appealing target for the development of interventions to promote improvements in health. However, more research is needed before we can effectively target sirtuins for therapeutic purposes. So, currently, although sirtuins represent promising therapeutic targets, their role in the regulation of mammalian lifespan remains an open question (Accili et al., 2011). Then, the future perspective could be represented by studies performed to identify the efficacy of sirtuin activators in the prevention and/or treatment of cardiovascular diseases such as heart failure.

### Conflict of interest statement

The authors declare that the research was conducted in the absence of any commercial or financial relationships that could be construed as a potential conflict of interest.
